# NLP-based music processing for composer classification

**DOI:** 10.1038/s41598-023-40332-0

**Published:** 2023-08-14

**Authors:** Somrudee Deepaisarn, Sirawit Chokphantavee, Sorawit Chokphantavee, Phuriphan Prathipasen, Suphachok Buaruk, Virach Sornlertlamvanich

**Affiliations:** 1 Sirindhorn International Institute of Technology, Thammasat University, Pathum Thani, 12120 Thailand; 2 Faculty of Engineering, Thammasat University, Pathum Thani, 12120 Thailand; 3 Asia AI Institute, Faculty of Data Science, Musashino University, Tokyo, 135-8181 Japan

**Keywords:** Computer science, Information technology

## Abstract

Categorizing music pieces by composer is a challenging task in digital music processing due to their highly flexible structures, introducing subjective interpretation by individuals. This research utilized musical data from the MIDI and audio edited for synchronous tracks and organization dataset of virtuosic piano pieces. In this work, pitch and duration were the musical features of interest. The goal was to innovate an approach to representing a musical piece using SentencePiece and Word2vec, which are natural language processing-based techniques. We attempted to find correlated melodies that are likely to be formed by single interpretable units of music via co-occurring notes, and represented them as a musical word/subword vector. Composer classification was performed in order to ensure the efficiency of this musical data representation scheme. Among classification machine learning algorithms, k-nearest neighbors, random forest classifier, logistic regression, support vector machines, and multilayer perceptron were employed to compare performances. In the experiment, the feature extraction methods, classification algorithms, and music window sizes were varied. The results were that classification performance was sensitive to feature extraction methods. Musical word/subword vector standard deviation was the most effective feature, resulting in classification with a high F1-score, attaining 1.00. No significant difference was observed among model classification performances.

## Introduction

Music has played a significant role in society and culture throughout human history. Our ancestors began producing music in a way that mimics natural sounds for religious and entertainment activities. While there is still a debate regarding whether music began with vocalization or the rhythmic pattern from anthropoid motor impulse, many believe that the human voice and percussion are ones of the earliest instruments to create human-made music^1^. On another note, rhythm is an essential element of music that can be generated independently^2^. Archaeologists have also discovered various primitive instruments, such as flutes, dating back over 35,000 years ago^3^. From the past to the present, the invention of music theory and idealization has evidently developed, giving rise to unique musical compositions. During the Renaissance, composers provided the basis that eventually became the Baroque style. Baroque composers began writing music for more sophisticated bands, which later evolved into full orchestras^4^. Bach and Vivaldi were undoubtedly outstanding composers of this age. Many composers in the classical era, most notably Mozart and Beethoven, experimented with various forms, melodic ideas, and instruments resulting in the creation of various noteworthy and well-regarded musical pieces such as Piano Sonata No.11 in A, Requiem Mass in D minor, Fidelio, op.72, etc. Romantic music, brought on by Chopin, Schumann, Brahms, and many others, is marked by emotional expression with musical dynamics^5^.

For centuries, humans have unceasingly developed music theory to gain a better understanding of music, ranging from notation defined for representing each sound to formalizing the rules and principles for arranging those sounds. Hence, humanity continually acquires many more rigid foundations for music comprehension. Notwithstanding, some gaps cannot be filled solely by the ground of traditional music theory. The “circle of fifths,” one of the essential concepts governing music theory, provides a guideline to organize the combination of musical notes. For example, C and G notes harmonize well together, making listeners emotionally pleasant. However, the circle of fifths cannot explicitly demonstrate the reason underlying such phenomena. Therefore, associating the music theory with scientifically measurable quantities is desired to strengthen the understanding of the nature of music. Pitch in music theory can be described as the frequency in the scientific domain, while dynamic and rhythm correspond to amplitude and varied duration of notes and rests within the music waveform. Considering notes C and G, we can also explore the physical rationale behind their harmonization. The two notes have integer multiples of their fundamental frequencies close to each other. For instance, note C in the fourth octave has a frequency of 523 Hz, and note G in the same octave has a frequency of 784 Hz, approximately. A double frequency of such 784 Hz, yielding the results of 1568 Hz, is relatively close to threefold of 523 Hz^6^. Thus, the physical implication of music theory is an important step toward a genuine comprehension of music.

In these modern days, there are attempts to recognize styles and composers of music, especially by applying a vast variety of computational techniques. The development of statistical machine learning and deep learning plays a crucial role in capturing insight into information underlying music. Well-known methods include the forced classification of music according to its composer or style categories. Theoretically, algorithms should be able to recognize patterns of musical data based on digital acoustic features. Music genre recognition, specifically obtained from Western, Latin American, and African music, based on spectrogram has been successfully explored using convolutional neural network (CNN) and support vector machines (SVM) classifiers^7^. Classification work on music composers and genres has been previously published by Bergstra et al. to predict these two identities using the ensemble learner Adaptive Boosting (AdaBoost)^8^. Deep learning has recently been widely applied for analyzing music, particularly for solving classification problems and music generation of different styles. Music time sequence and semantic information are gradually provided to the classifier. For example, Kim et al. classified 13 classical composers using a symbolic domain and audio features, yielding a satisfying result with an F1-score of 0.8333^9^. They proposed a framework that categorizes composers from segments of music pieces, focusing on note-related characteristics, e.g., pitch and note duration. This work offers additional insight into the usage of symbolic representation, which contributed to the motivating idea for our research.

Although some research was carried out on music composer classification, there were only a few investigations into musical features. Therefore, we examine the significance of acoustic features in the classification of music composers. Our previous attempt was to visually represent music by encoding acoustic features into pixels, creating 2-dimensional images of music. Composer classifiers trained using the visual representation achieved an F1-score of 0.85^10^. Alternatively, the acoustic attributes, including pitch and duration, obtained from MIDI pieces are converted into symbolic representation^11^ where features were processed using the *n*-gram technique in a similar manner to the text data analysis. Further statistical observations can then be performed. This leads to our work proposed in this paper, where the main aim is to validate the conceptualization of musical data representation by adopting natural language processing (NLP) techniques, especially applying word/subword segmentation to extract key acoustic-related feature vectors, contributing to the improved classification performance. The demonstration was carried out on an adjusted classical music dataset containing a collection of 809 music pieces composed by five composers who authored more than 100 music pieces, enabling suitable class sizes to train classification models. Machine learning algorithms, including k-nearest neighbors (kNN)^12^, random forest classifier, logistic regression, support vector machines (SVM)^13^, and multilayer perceptron (MLP), were utilized in the experiments classifying the music pieces by composers. We believe that the success of the composer classification relies on the performance of the musical data representation technique proposed.

This paper presents a novel approach to musical composer classification using an NLP-based data representation technique. The background part presents the concepts of word/subword segmentation, which can be applied in musical means. The information about the music dataset used in this work, the feature extraction methods, and the classification models used are discussed in the methodology. Then, the experimental results obtained from varying a set of parameters such as feature extraction methods, classification algorithms, and music window sizes are reported and discussed, along with suggestions for future work. Finally, the work is summarized in the conclusion.

## Background

This section outlines the natural language processing (NLP) techniques that may well be applied in musical data representation and classification, including word segmentation and tokenization. The background for musical data digitization is also discussed. The possibilities of developing a novel NLP-based method to extract feature vectors associated with each music piece are explored to discover numerical signatures representing individual composers.

### Word representation

Since the computation is accomplished by computing numerical data, a vector of real numbers is required as input to a calculation in general NLP tasks. *One-hot* encoding, in which each word is related to a *V*-dimensional vector, provides a straightforward word representation. Hence, *V* defines the vocabulary size. This simple approach places a “1” on the $\textit{i}$th position of each word and a “0” on the rest of the positions. Another method is to use *word embedding*, a distributed representation of text data that forms an *N*-dimensional real-valued vector for every word. This enables words with similar meanings to be encoded with similar vectors, resulting in a more compact representation that requires less processing resources and time. Mikolov et al.^14^ introduced an approach to learning the word embeddings called *Word2Vec*, which is trained based on neural networks.

*Word2Vec* can be obtained using two models, which are Continuous-Bag-of-Words (CBOW) and the Continuous Skip-Gram, to learn the word embedding. Both models are interested in identifying relevant information about words in the surrounding contexts, with a certain window of neighboring words. This window size is an adjustable model parameter. While the CBOW model uses the context to predict a current word, the skip-gram model uses the current word to predict its context^14^.

### Word tokenization

Word tokenization, also known as word segmentation, is a popular technique for working with text data that have no clear word boundaries. It divides a phrase, sentence, or whole text document into units of meaningful components, i.e. words. Each small unit resulting from text splitting is called a token.

To avoid the challenge of maintaining an up-to-date dictionary for dictionary-based word segmentation, statistical-based methods have been proposed. A previous study reported that the frequency of an arbitrary string drops as the length of the string is increased^15^. This is because as the length of the string increases, the number of possible combinations of characters also increases, making it less likely for any one particular string to appear with the same frequency as before. Moreover, it is observed that when any given string possesses more characters (within a possible word length), the occurrence frequency of such string significantly decreases. By combining this evidence of frequency dropping with the probability of co-occurrence between possible pairs of word strings, it is possible to identify the most likely word strings.

*SentencePiece* is an Apache license open-source software that performs as a language-independent unsupervised tokenizer and detokenizer for subwords^16^. It uses both the byte-pair-encoding (BPE)^17^ and the Unigram language model^18^ for its segmentation algorithm. The tokenization is carried out based on the frequency of character sequences, including white spaces. The input text is treated practically as a series of Unicode characters, and the white space is implicitly included as a normal character during the tokenization process by SentencePiece. The algorithm automatically replaces white spaces with the “_” (U+2581) character in order to elicit the behavior of all textual characters and determine co-occurring characters.

The BPE is an example of an advanced tokenization technique for neural machine translation (NMT) that encode words into subwords to compress pieces of information carried in text^19^. Technically, the BPE relies on a pre-tokenizer that obtains a list of subwords by slicing the sentence into individual characters and sequentially combining the most frequent neighboring pairs of those characters until the selected vocabulary size is reached. Vocabulary size must be defined prior to training the tokenizer. Note that the most effective way to extract subword sequences from a sentence is to consider its context, not to strictly define the same token to the same spelling vocabulary. However, the BPE algorithm only produces one unique segmentation for each word; thus, the probability of the alternative segmentation is not provided. This makes it difficult to apply the subword regularization technique, which requires the probability of alternative segmentation.

An alternative tokenization technique, the Unigram language model can generate considerable subword segmentation with probabilities. Kudo^18^ proposed this approach with a supporting assumption that each subword occurs independently. The probability of a subword sequence, $P(\textbf{x})$ is therefore easily expressed using the product of the probability of an individual subword occurrence, $p(x_{i})$ probabilities: For all $x_{i} \in V$; *i* = 1, ..., *M*,

1 $$\begin{aligned}{} & {} P(\textbf{x}) = \prod _{i=1}^{M} p(x_{i}), \end{aligned}$$

2 $$\begin{aligned}{} & {} \sum _{x_i \in V} p(x_{i}) = 1, \end{aligned}$$

where ${\textbf {x}} = (x_{1},\ldots , x_{M})$ is a candidate subword sequence that follows a specific order from the first subword up to the *M*th subwords, *V* is a set of pre-determined vocabularies and *i* is the index of the subword contained in the sequence. Following that, the most logical segmentation, ${{\textbf{x}}}^{*}$ for the sentence, *X* is determined by the maximum probability of a specific sequence of subwords that is most commonly occurs in a sentence: For ${\textbf {x}} \in S(X)$,

3 $$\begin{aligned} \textbf{x}^{*} = \text {argmax}(P(\textbf{x})), \end{aligned}$$

where *S*(*X*) represents a set of all possible sentence segments derived from *X*. These sequences of subwords are given a maximum probability by the Viterbi algorithm^20^, which yields the value for ${\textbf{x}}^{*}$. There is evidence indicating that the Unigram language model fits better with the nature of languages and subword sequencing behavior providing probabilistic interpretation. Additionally, it can return multiple segmentations along with their probabilities, which is necessary for subword regularization.

Overall, the unigram probabilities and the training corpus can theoretically be used to build SentencePiece on any Unigram model^16^. A suitable vocabulary size for the Unigram model parameters is adjusted using the Expectation–Maximization algorithm until the optimal loss in terms of the log-likelihood is achieved. The Unigram algorithm always preserves the base letters to enable the tokenization of any word. In addition to collecting the vocabulary, Unigram also saves the likelihood of each token in the training corpus so that the probability of any tokenization can be calculated after training, which allows it to choose the appropriate token.

### Musical data digitization

Through decades of advancement in music technology and digital transformation, there crystallized the two foundational unique yet harmonizing approaches to conveying music information in a digital environment as follows.

*Acoustic signal representation* An audio signal format can either be passed through compression, such as mp3 format, or preserved as pulse-code modulation (PCM), such as the waveform audio file format (WAV).

*Digital symbol configuration* Nowadays, several digital symbolic representations of music are accessible for use. Take as an example the MIDI, MusicXML, and ABC Music notation.

By comparing the two digital representations of music, they elucidate the noteworthy distinguishable characteristics as such. The symbolic representation can demonstrate and delineate the conception of music theory more unblemished in contrast with the acoustic signal, which does not explicitly impart the music theory, as it represents solely the voltage intensity over time. Furthermore, the audio recording may also incorporate insignificant background noise from the recording process. Another distinction between the two representations lies in the continuity of the information stream. Clearly stated, despite undergoing the sampling process, the audio signal representation nonetheless appears continuous. On the other hand, the symbolic representation deems a collection of discrete occurrences^21^. While continuity disparity directly correlates with computing, processing, and storing costs, implying that signal representation requires more resources and storage than the symbolic medium. Moreover, dealing with the signal representation directly requires an added layer of complexity in order to extract meaningful acoustic features. For these reasons, the digital symbolic configuration, precisely the MIDI format, is considered more desirable for our work.

### Potential of applying NLP-based techniques for interpretation of music pieces

Unlike text articles, determining an actual meaning in instrumental music is challenging since it lacks a clearly interpretable structure. Kinds of musical grammar are developed based on the theories associated with the music eras or genres yet subjective to individuals in how it was formed and how it sounds. Nonetheless, both languages and music pieces are products of human creations. Such creations aim to communicate pieces of information between persons which should be understood by humans. For languages, direct interpretation can be communicated from person to person with a mutual understanding of definitions of words and sentence structure. In contrast, there is no agreed standard to determine music’s meaning. However, music can be communicated from the composer via performance to the listener, sending meaningful pieces of information that can be perceived physically through ears and felt emotionally with melody or sequences of notes. These days, a large quantity of MIDI files are available. It is a widely used format for symbolically representing music pieces, and encoding commands for synthesizers and other studio devices, which are commonly associated with instrumental music. It is a versatile format for creating and sharing music compositions that may also include commands for synthesizing voice and other types of audio^22^. The information that MIDI files provide are note pitch, note duration, key striking velocity, etc. Given their intimate nature, exploring the ability of the NLP-based techniques, including word representation and tokenization, specifically Word2Vec and SentencePiece techniques, to understand music could pave the way to an alternative musical data representation and gain related interpretation.

## Methodology

This Methodology section describes the MAESTRO dataset, the proposed musical feature extraction methods, and the machine learning models used in the composer classification experiments as follows.

### MAESTRO dataset

The MIDI files utilized in this study were obtained from the *MAESTRO Dataset*^23^, which comprises over 200 hours of concert-quality piano performances amass over a decade of *International Piano-e-Competition*. This dataset includes very precise musical note alignments with less than 3 ms variation, as well as extra information on piano performance parameters such as note duration, piano-key striking velocities, and sustain/sostenuto/una corda pedal position. This information was recorded into a MIDI file by the high-precision MIDI capture and playback system embedded in the Yamaha Disklaviers piano used throughout the competition. Along with the exceptional quality MIDI files, the dataset contains related metadata such as the composition title, composer name, year of performance, and duration of each music piece. Previously, the maestro-v2.0.0 was presented as input, which has been partitioned to classify composer according to major voting of segment-wise prediction^9^. Furthermore, a large-scale symbolic music corpus was created by merging the maestro-v3.0.0 with other MIDI datasets in order to improve the pre-training model on four music understanding tasks, covering melody completion, accompaniment suggestion, genre classification, and style classification^24^.

**Figure 1:**
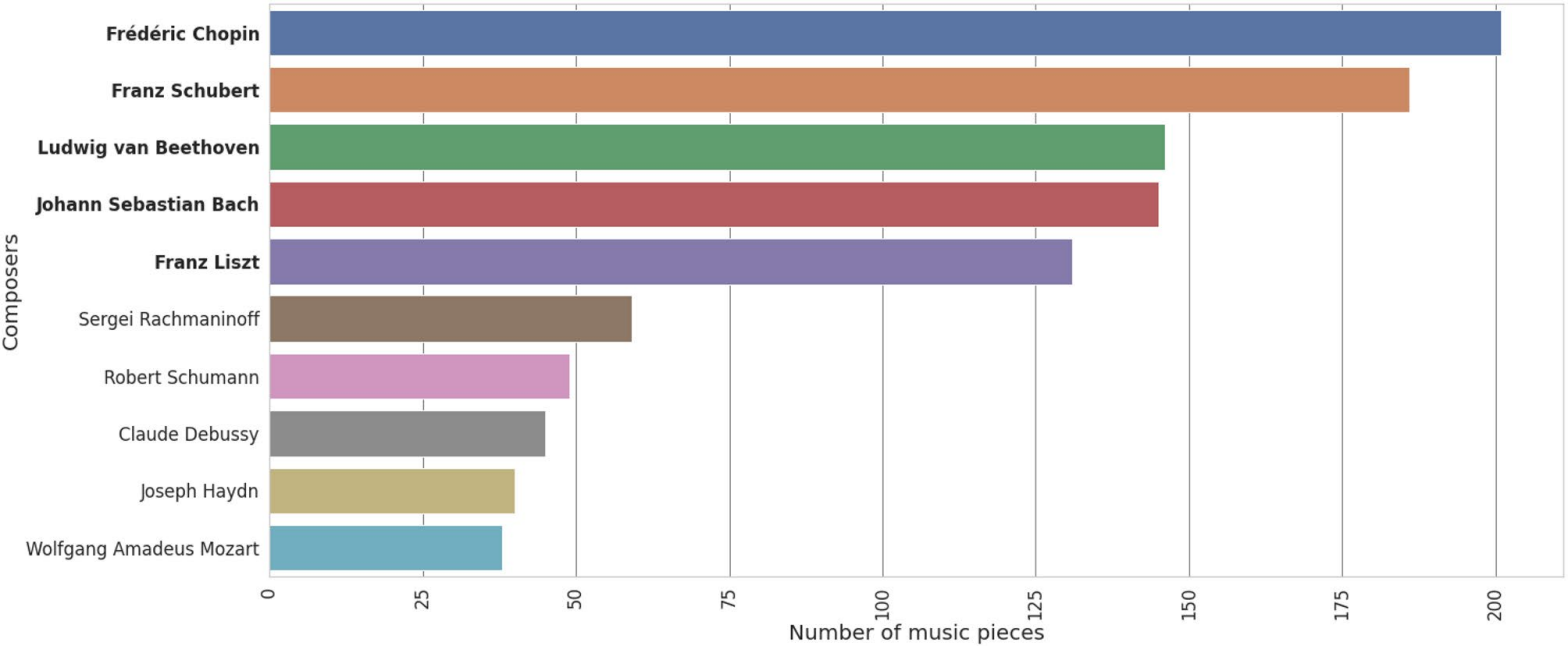
Number of music compositions composed by each composer in the *MAESTRO dataset* sorted in descending order.

In this research, the maestro-v3.0.0 was selected as the prominent dataset. It encompasses a total of 1276 piano arrangements by 60 distinct composers, which is equivalent to 198.7 hours of performances. Among these 60 composers, five composers who composed more than 100 music pieces in this dataset were chosen: Franz Liszt, Franz Schubert, Frédéric Chopin, Johann Sebastian Bach, and Ludwig van Beethoven, since they composed a comparatively significant number of piano compositions among composers included in the *MAESTRO Dataset* as illustrated in Fig. 1. Then, the data underwent the data-splitting process as described in the “Classification models” section.

### Feature extraction

Due to the continuous progression in the field of music information retrieval (MIR), various musical feature extraction tools for both audio signal representation and symbolic representation are available in the market. Since the data used in this study is in MIDI file format, which is a kind of symbolic music representation, thereafter, the scope of tools can be narrowed down. Amid these tools, one was standout pretty_midi^25^.

pretty_midi is a Python library for extracting musical features from symbolic music representation. It has been used throughout multiple study domains in relevance to music. For music composition, this Python library pretty_midi was used to excerpt the piano roll information from *Lakh MIDI dataset*^26^ and create *Lakh Pianoroll Dataset*, which was further processed and cleaned to make training dataset for MuseGAN, the generative adversarial network (GAN) for music generation^27^. In the dataset generation for music source separation, Manilow et al. also leveraged the pretty_midi library as a tool which contributed to the creation of *synthesized Lakh dataset*, the dataset that was specifically published for music source separation studies^28^.

The extraction process performed in this work begins by extracting crucial information, including note pitch, start time of each note, and end time of each note from each music piece using pretty_midi. Then, the start time and end time of each note are further computed to generate another feature, namely note duration. This process results in a coordinate pair representing each note as shown in Eq. (4). In this experiment, we encode only the note pitch and duration but exclude the key striking velocity from our representation. The first reason is that, by incorporating the velocity into the tuple, there will be a myriad of tuples hence characters in our vocabulary. This excessive number of characters in vocabulary may hinder the ability of the model to recognize the pattern. Another intuition can be observed from real-world circumstances. That is, considering only the notes being played and their duration, one can tell which piece it is or even who composed this piece based on their knowledge. For instance, suppose a bad pianist is playing the Piano Sonata No.11—Rondo Alla Turca by Mozart with the correct note and duration but without the appropriate dynamic in this sense, the variation in key striking velocity. The audience should still be able to recognize which piece is being played. Thus, we do not include the velocity information in our representation.

4 $$\begin{aligned} (P_i, D_i) = \left( P_i, T_{i_{end}} - T_{i_{start}}\right) , \end{aligned}$$

where *i* is the note index, *P* denotes the MIDI integer encoded pitch, and *D* denotes the duration (in seconds) of the note. $D_i$ is derived from the subtraction of note onset, $T_{i_{start}}$ from the note termination, $T_{i_{end}}$ of the *i*th note.

Note that we considered the polyphonic music piece as a whole without reducing it to only one channel. Hence, there will possibly be notes which co-occur. Contemplating the NLP aspect, each concurrently occurring note can be viewed as a concurrent character, which may be odd for Western languages. Nonetheless, the simultaneous occurrence of characters is relatively common in some Southeast Asian languages, such as Thai and Lao. Thus, Applying the NLP approach directly to polyphonic music with concurrency is reasonably practical. However, there is still a remaining issue, which is the procedure of ordering those co-occurring notes. Thereby, we introduce a rule for tie-breaking amid those notes utilizing the pitch of each of them. To clarify, we arrange the concurrent note tuples in descending order concerning the MIDI pitch value to ensure the consistency of the derived data.

**Figure 2:**
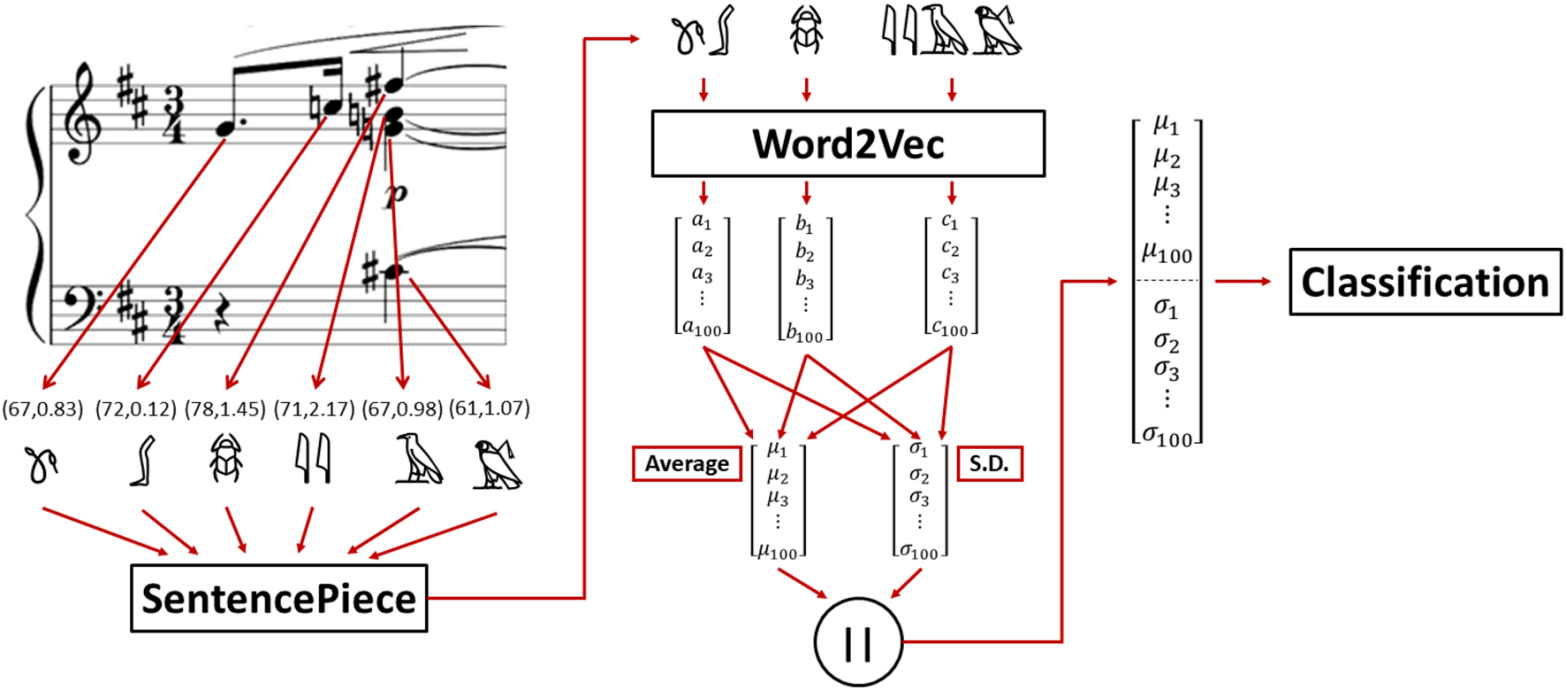
System diagram for the proposed NLP-based music feature extraction procedure.

After deriving all the tuples, they are mapped into arbitrary Unicode characters where the same character represents the same tuple. Then, these characters undergo the SentencePiece^16^ algorithm to group sequences of commonly occurring characters into words or subwords. For the music comparison, sequences of commonly occurring notes were determined at this stage. Next, we utilize the Word2Vec approach^14^ (previously described in the Background) to transform the musical words/subwords extracted from the SentencePiece step into a vector. After obtaining all the vectors for each musical word/subword, we finally derive a vector representative of each whole music piece by averaging those musical word vectors of that particular piece, concatenated with their standard deviation vector (SD) as shown in the following Fig. 2. Hence, we obtain the NLP-based music representation to be processed in the music composer classification task.

### Classification models

In this research, five classical machine learning models for classification, including k-nearest neighbors (kNN), random forest classifier (RFC), logistic regression (LR), support vector machines (SVM), and multilayer perceptron (MLP), were used to classify the music into one of the five groups according to its composers. The entire dataset was divided into train and test datasets to develop the model learning and evaluation. The ground truth labels of composers were reserved for training and testing purposes. The data-splitting was performed to evaluate the performance of classification models with the training:test ratio of 8:2. For all traditional machine learning techniques, except the MLP, the fivefold cross-validation was applied to the training dataset to evaluate the performance of training the machine learning classifiers. The training dataset was partitioned into five equal subsets, with a different subset used as the validation set in each iteration. For the MLP approach, the averaged F1-score was calculated from the validation set of 200 epochs in the model training step to ensure the parameters converge. The averaged validation F1-score for each model was obtained. This technique allows us to ensure the robustness and generalization of the model built. The F1-score was chosen as a performance metric to assess whether the classifiers possessed high precision and recall in correctly categorizing the music pieces into their five composers. This indicator gives significant weight to low values, which makes it a suitable evaluation metric for this kind of imbalanced classification problem.

## Results and discussion

In this work, two predominant approaches for feature extraction procedures were conducted, namely the extended Wolkowicz method and our proposed NLP-based method. Our proposed method involves three variations utilizing the representative musical word/subword vectors underlying music pieces: average (Avg), standard deviation (SD), and average concatenated with standard deviation (Avg + SD). Initially, we adopted the feature extraction process from Wolkowicz et al., which acquires the tuple from the relative distance between the pitch of consecutive notes and the logarithmic ratio between its duration to gain a definition of individual musical characters—i.e. notes. With our extension by utilizing the SentencePiece, we were able to split the piece of music into the common occurrence musical word/subword and then applied Word2Vec to derive those word vector representatives within each piece. Then, the average among the vectors for each music piece was obtained to generate a summarized vector of that particular piece. By exploiting this technique with a skip-gram of window size of 9, the highest F1-score of 0.456 was achieved on the test dataset using the kNN model for classification. The hyperparameter tuning technique was applied in order to observe the upper limit of its classification performance. PyCaret framework^29^ was used as a tool to perform 50 iterations of random hyperparameter tuning. The fine-tuned model was then utilized to make predictions on the same test dataset, resulting in an F1-score of 0.371, which was not improved from the original test result. Whereas our proposed procedure uses all variations of musical word/subword representation, including Avg, SD, and Avg + SD, obtaining the maximum F1-score of 0.538, 1.00, and 1.00, respectively, as shown in Table 1.

It is clearly seen that our proposed methods outperform the extended Wolkowicz method. Especially, when the standard deviation of the musical word/subword vectors is incorporated, exceptional results are obtained. It was suggested that musical notes correspond to the level of word structure as in the NLP representations^11^. This conceptual idea was presented in their data transformation process that extracts the relative distance between consecutive notes calculated from the numerical representation of pitch and duration. Nevertheless, the idea of using only the relative distance between consecutive tokens does not convey much meaningful information from the NLP perspective because it only focuses on the relative difference between consecutive notes within the music but lacks precise note information at each instant.

We, therefore, propose a novel relationship paradigm between natural language structure and musical organization as in Table 2 with a modified strategy for obtaining the tuples. In conformity with our paradigm, the derived tuple denotes a character from the NLP’s viewpoint by acquiring the information directly from each note’s characteristics. Hence, it is logical to apply the NLP techniques, namely, SentencePiece, which consolidates notes into groups of notes that mutually appear together in a similar manner with defining commonly occurred characters into words/subwords, and then applies Word2Vec to generate vector representatives of those groups of notes, respectively. The closeness of the groups of notes in terms of both note characteristics and the variation of notes within a piece of music should infer the fingerprint of each music composer. These can be adopted as a ground for the musical data representation which is specifically applied in this work for composer classification.

**Figure 3:**
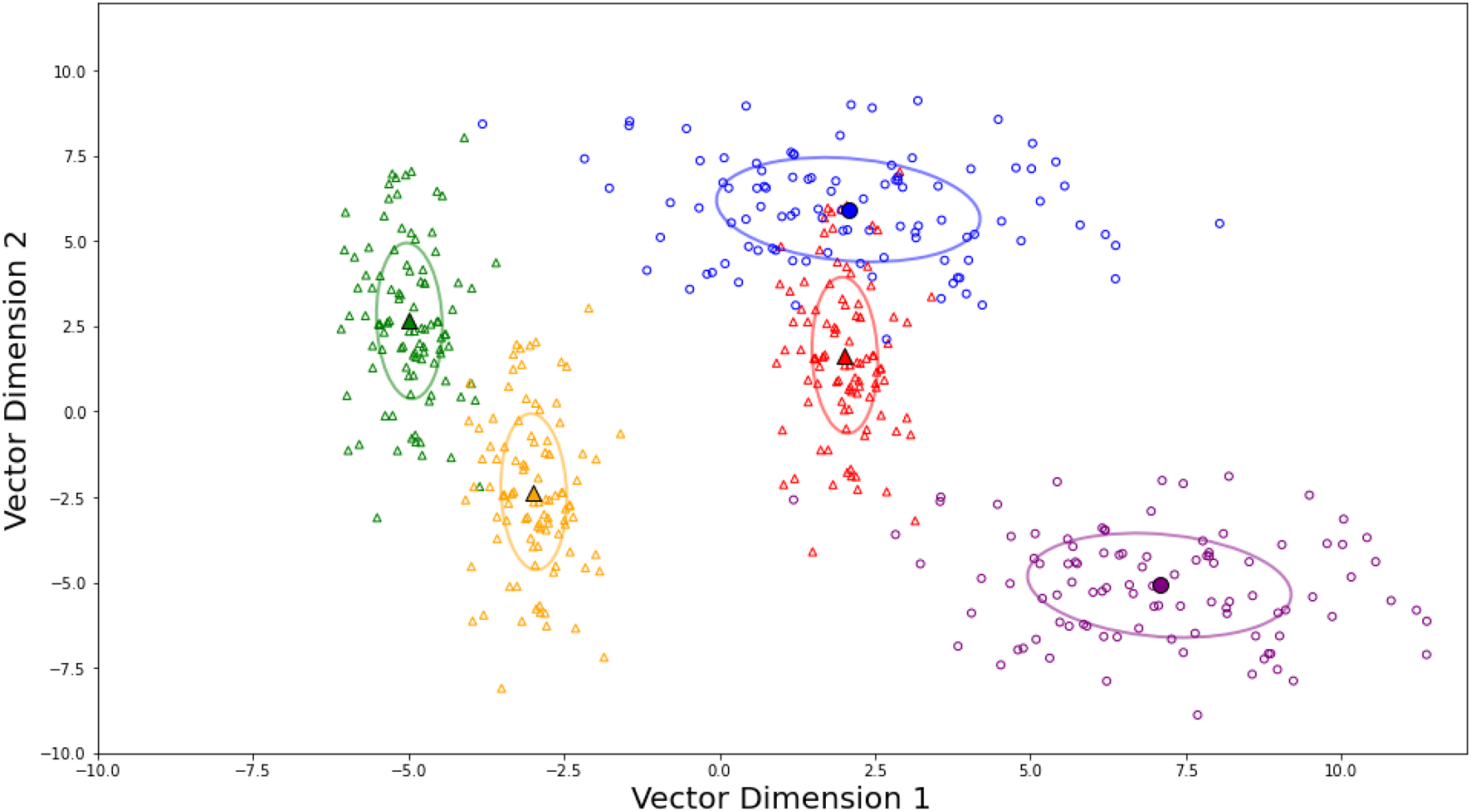
Schematic diagram of musical word/subword vector plotted component-wise in 2-dimensional arbitrary space: distinct colors denoted different music pieces, different marker shapes correspond to different composers. For each music piece, the huge solid marker with a black edge depicts the average musical word/subword vector of the music, and the ellipse represents the standard deviation of the musical word/subword vector.

To elaborate the results, the maximum F1-score for composer classification when utilizing our feature extraction approach with the use of the averaged musical word vectors of music pieces only spikes up to 0.538 using the RFC model with a skip-gram of window size equal to 7 as shown in Table 1. This scheme outperforms the highest classification performance of the extended Wolkowicz approach by roughly 8 percentage points. The dataset of averaged word vectors was fine-tuned, similar to the extended Wolkowicz case, which resulted in an F1-score of 0.404 on the test dataset, lower than the original test result, possibly due to randomization However, the standard scheme using the average vector to represent each music piece can capture only certain information, which is the usage of groups of notes but the numerical representation highly depends on particular keys. On the other hand, there could potentially be useful information, such as the distribution of words, which should also be included in the analysis. Thus, adding information on the distribution of words, i.e. the dispersal of notes, should contribute to a better understanding of music pieces. That is, composers do not restrict their music arrangement to a particular key, but how they compose the variety of notes within the music is rather more important to signify their fingerprints, conforming with the concept of relative representation in which the relative pitch to the first note is used to capture composer’s characteristic. For example, the 48 preludes and fugues composed by Johann Sebastian Bach consist of 24 preludes and 24 fugues from every existing key^30^. Given the characteristic of this particular composer, the average notes vary vastly from music to music, whereas the sizes of note diversity are similar among his music pieces. Hence, we propose the use of the musical word/subword standard deviation (SD) vector, which represents the distribution of notes, to create the musical representation vector by concatenating with the average vector (Avg + SD vector) and solely on its own (SD vector). The results from this procedure conform with our intuition since the highest F1-score skyrocketed to 1.00 for several combinations of models and skip-gram’s window size when comprising standard deviation into the representation vector. Furthermore, our investigations unravel additional astonishing insights. The result signifies that the sole standard deviation (SD) vector is adequate for providing necessary information to the model, even better, pushing more combinations of models and window sizes toward an impeccable result of a 1.00 F1-score. No significant difference in classification performance was observed among the various models built. This implies that standard deviation (SD) vectors are a phenomenal representation of the composer’s characteristics. Figure 3 illustrates an example scenario in which there were music pieces composed by two different composers denoted by triangle and circle markers. Two different music pieces belong to each one of the composers, represented by different colors, green and orange for the first composer corresponding to triangle markers, and blue and purple for the second composer corresponding to circle markers. It is supposed that there is an unknown music piece denoted by red color. If we would like to classify whether this music is composed by the first or the second composer, without any additional information, we can either consider the average vector of that music, the standard deviation of it, or the average concatenated with the standard deviation vector. Taking into account only the average vector in Fig. 3, we may conclude that the questioned music should belong to the second composer, who is represented by circle markers. In contrast, if the standard deviation vector which is demonstrated by the ellipse contour is considered, we shall interpret that this music belongs to the first composer whose markers are triangles.

In addition, even if the extended Wolkowicz and averaged word vector models undergo fine-tuning, they still perform far worse than the proposed method. Where the proposed method, which takes into account the standard deviation vectors, can give outstanding results without the need for a fine-tuning application. Hence, we can conclude that our proposed data representation gives such impactful features for classification that all machine learning models built are robust and generalize well in training and test datasets, with parametric flexibility. The validation F1-scores for the traditional machine learning models, including kNN, RFC, LR, and SVM, were calculated using the fivefold cross-validation, while 200 epochs were required to train and validate MLP models. The F1-scores obtained from the testing dataset align well with the validation dataset, as seen in Table 1 for all classifiers. This assures the non-overfitted models, particularly for our proposed standard deviation of the musical word/subword vectors approach where test and validation performance are close. Moreover, the results show that both the traditional machine learning models and MLP models exhibit comparable performance when evaluated using our proposed standard deviation vector approach. This confirms that the data representation is robust and can be used with confidence for any classification model.

**Table 1:** The F1-scores for 5-composer classification using each feature extraction method with varying machine learning models and skip-gram window sizes were evaluated on both the test dataset and the validation set (shown in parentheses).

Feature extraction method	Model	Window size						
		3	4	5	6	7	8	9
Extended Wolkowicz^11^	kNN	0.422 (0.422)	0.430 (0.399)	0.435 (0.436)	0.447 (0.420)	0.401 (0.405)	0.440 (0.425)	**0.456** (0.417)
	RFC	0.399 (0.467)	0.389 (0.453)	0.411 (0.459)	0.419 (0.453)	0.417 (0.476)	0.408 (0.482)	0.402 (0.470)
	LR	0.349 (0.371)	0.337 (0.377)	0.366 (0.380)	0.339 (0.389)	0.352 (0.386)	0.349 (0.392)	0.337 (0.396)
	SVM	0.351 (0.409)	0.349 (0.410)	0.329 (0.444)	0.372 (0.426)	0.324 (0.420)	0.331 (0.417)	0.324 (0.422)
	MLP	0.334 (0.238)	0.337 (0.234)	0.350 (0.259)	0.327 (0.260)	0.327 (0.229)	0.381 (0.254)	0.345 (0.276)
Avg	kNN	0.439 (0.332)	0.486 (0.396)	0.456 (0.382)	0.516 (0.426)	0.476 (0.413)	0.522 (0.467)	0.523 (0.451)
	RFC	0.456 (0.406)	0.502 (0.436)	0.511 (0.453)	0.512 (0.471)	**0.538** (0.476)	0.532 (0.477)	0.526 (0.487)
	LR	0.452 (0.362)	0.366 (0.354)	0.468 (0.343)	0.418 (0.365)	0.428 (0.397)	0.428 (0.391)	0.499 (0.400)
	SVM	0.349 (0.375)	0.449 (0.385)	0.436 (0.374)	0.436 (0.386)	0.488 (0.396)	0.507 (0.405)	0.475 (0.403)
	MLP	0.438 (0.264)	0.362 (0.249)	0.365 (0.270)	0.413 (0.293)	0.429 (0.305)	0.416 (0.272)	0.428 (0.307)
SD	kNN	0.994 (0.997)	0.994 (0.995)	**1.00** (0.997)	0.994 (0.995)	0.994 (0.995)	0.994 (0.995)	**1.00** (0.997)
	RFC	**1.00** (1.00)	**1.00** (0.997)	**1.00** (0.998)	**1.00** (0.998)	**1.00** (0.998)	**1.00** (0.998)	**1.00** (1.00)
	LR	**1.00** (0.997)	**1.00** (0.997)	**1.00** (0.997)	0.994 (0.997)	**1.00** (0.997)	0.994 (0.997)	**1.00** (0.997)
	SVM	0.982 (0.988)	0.982 (0.988)	0.982 (0.989)	0.988 (0.989)	0.988 (0.991)	0.988 (0.989)	0.982 (0.988)
	MLP	**1.00** (0.971)	0.990 (0.976)	0.995 (0.976)	0.979 (0.971)	0.995 (0.975)	0.990 (0.971)	0.997 (0.977)
Avg + SD	kNN	0.988 (0.975)	0.988 (0.981)	0.981 (0.978)	0.994 (0.978)	0.981 (0.981)	0.988 (0.978)	0.988 (0.981)
	RFC	**1.00** (0.998)	**1.00** (0.997)	**1.00** (0.998)	0.994 (0.997)	**1.00** (1.00)	0.998 (0.998)	**1.00** (0.998)
	LR	**1.00** (0.988)	**1.00** (0.992)	**1.00** (0.991)	0.994 (0.995)	**1.00** (0.989)	**1.00** (0.988)	**1.00** (0.994)
	SVM	0.963 (0.974)	0.982 (0.983)	0.988 (0.974)	0.982 (0.974)	0.988 (0.977)	0.975 (0.980)	0.988 (0.977)
	MLP	0.976 (0.949)	0.984 (0.952)	0.984 (0.956)	0.990 (0.949)	0.984 (0.944)	0.969 (0.952)	0.995 (0.961)

For the four traditional machine learning models, (kNN, RFC, LR and SVM), the validation F1-scores were calculated using fivefold cross-validation. For the MLP, the averaged F1-scores were calculated from the validated F1-score of 200 epochs of training.

The highest F1-scores among each feature extraction method are in [bold].

Apart from the 5-composer classification, we also extended our experiment to classify up to 14 composers bestowing the result of 1.00 F1-Score for both kNN and random forest classifier model utilizing skip-gram of window size equal to 6 and SD musical word/subword vector representation. Where each composer composed more than or equal to 25 music pieces. This confirms and validates our composer classification pipeline using the proposed NLP-based music data representation approach.

**Table 2:** NLP and music relationship paradigm; modified from Wolkowicz et al.^11^.

Linguistic levels	Natural language	Music
Phonetics	Voice audio	Piano pieces
Phonology	Phonemes	Sound of each notes
Morphology	Character/word	Note/group of notes
Syntax	Ordering of words	Notes arrangement
Semantics	Meaning	Diatonic function
Pragmatics	Practical usage of language	Musical phrase

This work innovates the novel statistical-based musical data representation toward gaining musical interpretation, which is successfully demonstrated via solving the composer classification problem. Currently, the frequent co-occurring notes can be captured by statistics. The remaining curiosity is to discover the connection between machine and human intelligence. This may be addressed by the application of machine learning transformer techniques, such as Dis-Cover AI Minds to Preserve Human Knowledge^31^ (i.e., the music theories, structures, and concepts that produce common co-occurring notes that form naturally-sound music as played by expert musicians) in a similar way that it applied to natural language grammar. A concrete interpretation of musical data can potentially contribute to advancing music generation and recommendation technologies.

## Conclusion

This work manifested the possibilities of incorporating knowledge from the field of natural language processing (NLP) into music information retrieval and interpretation. In this study, musical features extracted from the combination of pitch and duration are interpreted as natural language grammatical structures. NLP techniques, word/subword tokenization using *SentencePiece* and word embedding using *Word2Vec*, were applied to extract co-occurring notes to be represented as a musical word/subword vector. These meaningful features underwent composer classification experiments. It was observed that the main characteristic that signified the composer’s fingerprints was the variety of notes used within a music piece. Hence, the 5-composer and 14-composer classifications using musical word/subword standard deviation vector achieved the F1-Score of 1.00 in various classification models. The proposed scheme not only grants outstanding results for composer classification, but it is also the foremost stepping stone toward a thorough comprehension of this intriguing invention of humanity, the music.

## Data Availability

The data that was used to carry out this research is publicly available from https://magenta.tensorflow.org/datasets/maestro.

## Code availability

The computer code used for conducting the experiments and generating results can be accessed from https://github.com/SirawitC/NLP-based-music-processing-for-composer-classification.
